# Supplementary material for: Colorectal Cancer Survival Prediction Using Deep Distribution Based Multiple-Instance Learning
Source: Entropy (Basel). 2022 Nov 15;24(11):1669. doi: 10.3390/e24111669 (PMC9689861; doi:10.3390/e24111669)
Supplement: Supplementary file 1 [file entropy-24-01669-s001.zip › entropy-1985218-supplementary.pdf]

Supplementary Figure:

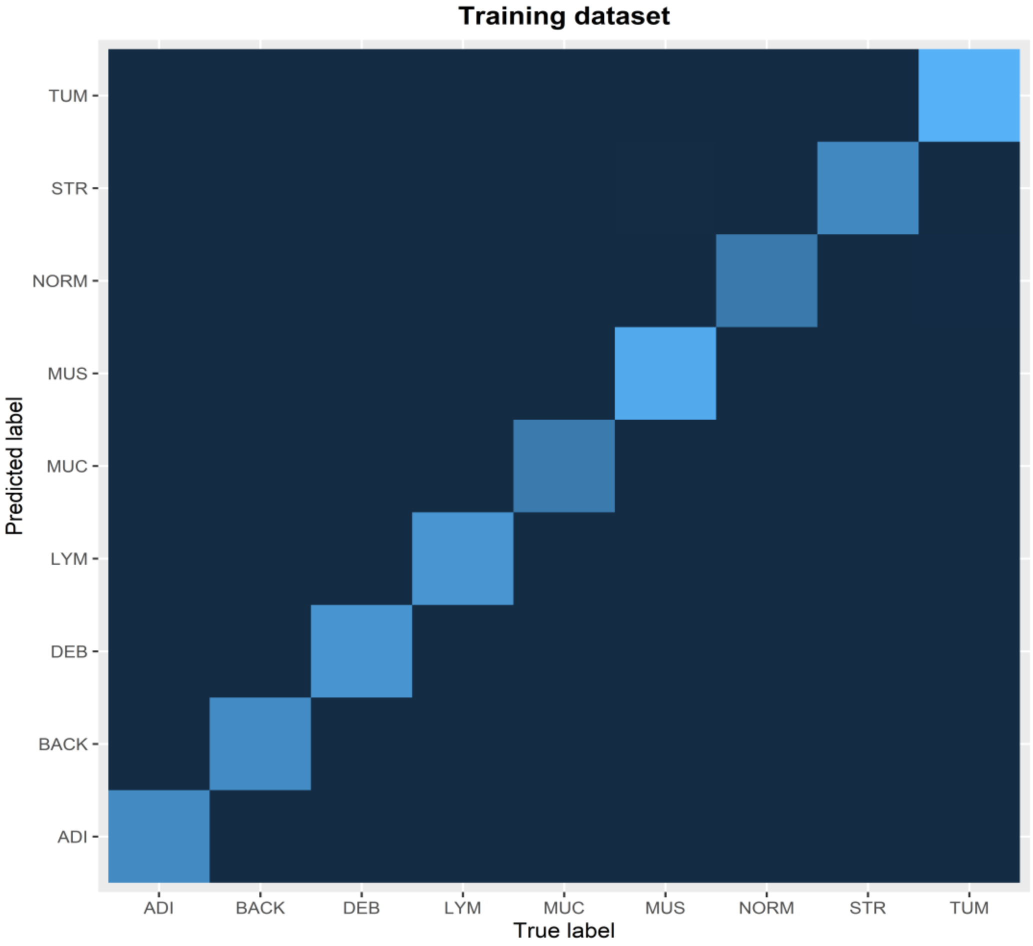

(**a**)

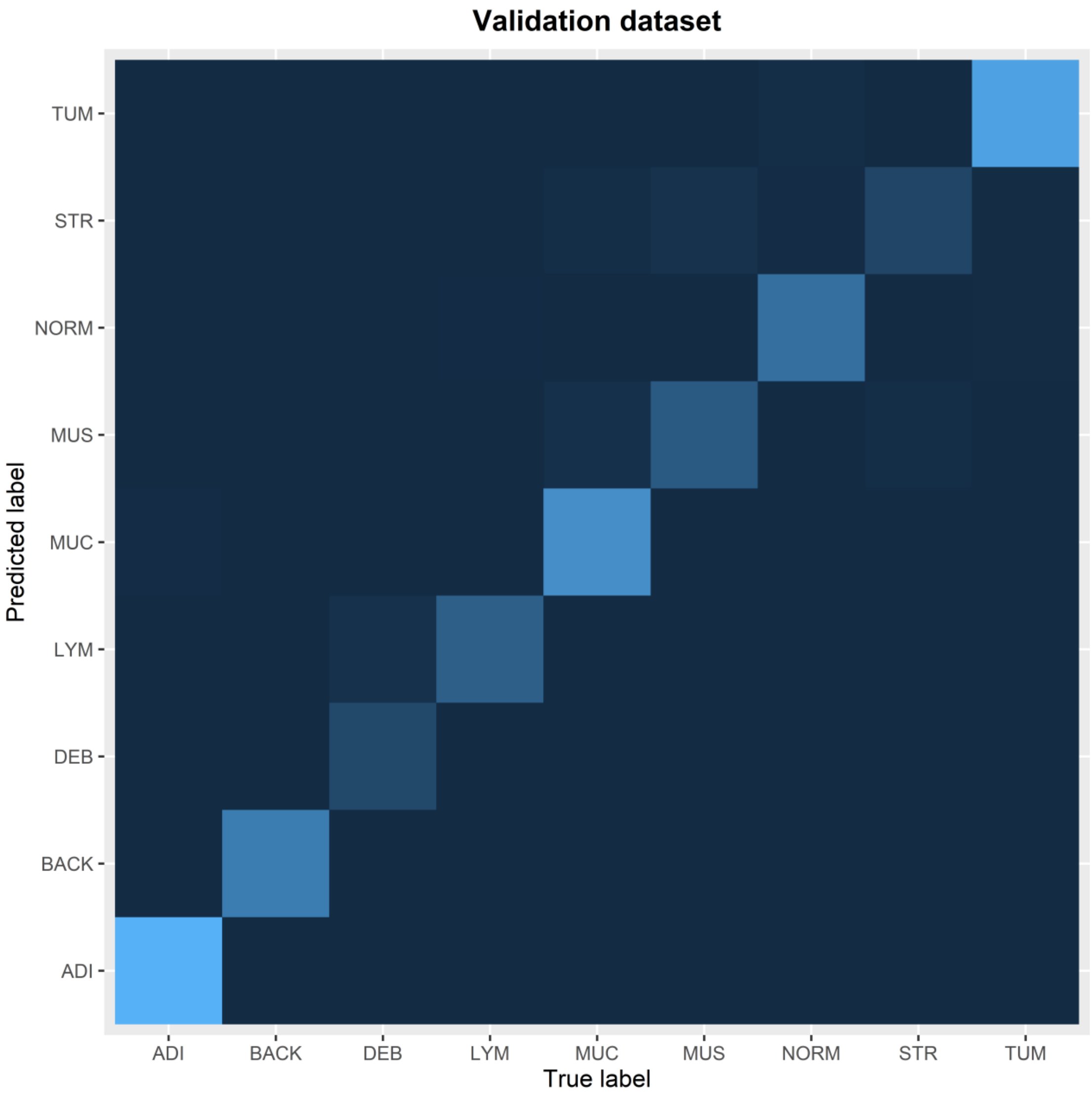

(**b**)

Figure S1. The overall accuracies of the tissue-type classification model were 99% on (a) the training dataset NCT-CRC-HE-100K and 94.4% on (b) the validation image set xxx, respectively.
